# CRFR1‐Antagonism Ameliorates Early AD Pathology and Alters Stress‐Induced Changes in Pathology

**DOI:** 10.1002/alz.092978

**Published:** 2025-01-03

**Authors:** Maya Anne Ellisman, Floyd Sarsoza, Robert A. Rissman

**Affiliations:** ^1^ University of California, San Diego, La Jolla, CA USA; ^2^ Veterans Affairs San Diego Healthcare System, La Jolla, CA USA

## Abstract

**Background:**

Chronic stress has been linked to an increased risk for various health issues, including Alzheimer’s disease (AD). While novel therapies for AD have improved disease prognosis, a deeper understanding of the link between stress and AD may delineate potential novel preventative treatments. Within the stress system, corticotrophin‐releasing factor receptor 1 (CRFR1) has been shown to influence AD pathological hallmarks. Drugs targeting CRFR1 may elucidate how CRFR1 alters AD and provide a potential novel treatment approach.

**Methods:**

To examine the effects of CRFR1‐antagonism and chronic stress on AD pathology, young WT/PSAPP mice (6‐month) underwent treatment with vehicle/R121919 alongside chronic restraint stress. Then, subjects were assessed with a series of behavioral tests measuring learning, memory, and cognitive flexibility. Additionally, brain regions were immunohistologically analyzed for plaque deposition and biochemically analyzed for various Aβ species.

**Results:**

CRFR1‐antagonist treatment in early AD improves learning and cognitive flexibility, lowers plaque levels throughout the hippocampus and cortex, and lowers highly toxic cortical soluble Aβ oligomers while increasing insoluble Aβ monomers. Chronic restraint stress improves learning and memory, increasing plaques in the hippocampus and lowering cortical plaques while increasing insoluble cortical Aβ monomers. With CRFR1‐antagonism, stress‐induced benefits to learning are more pronounced, yet the stress effects on memory are abolished. At the same time, stress combined with CRFR1‐antagonism lowers the number of cortical plaques and insoluble cortical Aβ monomers, while increasing cortical Aβ soluble oligomers.

**Conclusions:**

CRFR1‐antagonism has the potential to improve and may be beneficial for stress‐induced AD pathogenesis in the early phases of AD.

